# Celiac disease and upper secondary school achievement in Sweden A retrospective cohort study

**DOI:** 10.1186/s12887-022-03773-6

**Published:** 2022-12-12

**Authors:** Katarina Johansson, Fredrik Norström, Peter H. R. Green, Anneli Ivarsson, Linda Richter Sundberg, Anders Själander, Anna Myleus

**Affiliations:** 1 Department of Public Health and Clinical Medicine, Umeå University, 901 87 Sundsvall, Sweden; 2 Department of Epidemiology and Global Health, Umeå University, 901 87 Umeå, Sweden; 3 Department of Medicine, Celiac Disease Center, Columbia University College of Physicians and Surgeons, Columbia University, New York, NY USA; 4 Department of Public Health and Clinical Medicine, Umeå University, 901 87 Umeå, Sweden

**Keywords:** School performance, Follow-up, Celiac disease, Grades, Gluten-free diet

## Abstract

**Background:**

Both undiagnosed celiac disease and some chronic childhood diseases are associated with lower academic achievement. However, there is little knowledge of achievements in those diagnosed with celiac disease. Our aim was to investigate school achievements in upper secondary school among Swedish adolescents with celiac disease.

**Methods:**

We performed a retrospective cohort study using register data. We analyzed choice of upper secondary school program, completion of upper secondary school including achievements of basic eligibility for college/university, and final grade in individuals with celiac disease diagnosed before 15 years of age, born 1991–97. We compared with the Swedish population of the same birth years. Analyses were adjusted for sex, year of birth, living region at 17 years of age, and parental education as well as income.

**Results:**

The cohort included 734 074 individuals, whereof 3 257 (62% females) with celiac disease. There was no significant difference in choice of upper secondary school program. No significant difference was found in completion or achieving basic eligibility for college/university in adjusted analyses. The mean final grade in the celiac disease group was 13.34 (standard deviation 4.85) compared to 12.78 (standard deviation 5.01) in the reference population (*p* < 0.001), out of a maximum of 20. The effect of celiac disease on final grade remained in adjusted analyses (*p* = 0.012).

**Conclusions:**

We found that diagnosed celiac disease does not negatively affect school achievements in upper secondary school. This finding suggests the diagnosis, treatment and follow-up programs of celiac disease could reverse potential deleterious academic processes.

**Supplementary Information:**

The online version contains supplementary material available at 10.1186/s12887-022-03773-6.

## Background

Celiac disease is an autoimmune disease that causes an immune-mediated enteropathy triggered by oral consumption of gluten [1]. The prevalence is around 1–3% [1, 2] and higher among females [1]. Celiac disease can cause a wide range of symptoms with both intestinal and extra-intestinal manifestation. Celiac disease is associated with an increased risk for other autoimmune diseases, like type one diabetes and autoimmune thyroid disease [1]. Untreated the disease is associated with an increased risk for other diseases like anemia, depression, lymphoma, and slightly increased mortality [1, 3]. Celiac disease is life-long and treated with a strict gluten-free diet [1]. Following a strict gluten-free diet can be challenging and previous studies have shown an adherence rate between 23–98% in children and 42–91% in adults [4, 5]. The expected effects of the treatment are mucosal healing with subsequent reduction in symptoms, serological markers, and co-morbidity [1]. Still, symptoms are common and often caused by voluntary and involuntary dietary lapses [6].

Several chronic conditions such as type one diabetes, epilepsy, and recurrent pain have been associated with poorer school achievements [7–9]. To our knowledge few previous studies have investigated celiac disease and school achievements in children and adolescents. Namatovu et al. performed a cohort study on Swedish children showing that school performance in ninth grade was not affected by celiac disease [10]. Similarly, an Italian cross-sectional study showed that participants with celiac disease passed high school and upper secondary school to the same degree as the regional reference data [11]. Undiagnosed celiac disease was not associated with lower academic achievements in upper secondary school, but differences were seen in higher education in a small Finnish study [12].

In Sweden, the progression of education is structured as nine-year compulsory school and after completion adolescents can apply for upper secondary school. Upper secondary school is a vocational and/or university preparing three-year education. Achievements in upper secondary school in Sweden affects eligibility and competitiveness when applying for higher education, or when looking for work, thus future career prospects [13]. Therefore, we aimed to investigate if celiac disease influenced the choice of upper secondary school program, the completion of upper secondary school, and the final grade from upper secondary school in Swedish adolescents.

## Methods

A nationwide register-based retrospective cohort study was performed.

### Register data

The Swedish Initiative for Research on Microdata in the Social and Medical Sciences 2.0 (Umeå SIMSAM lab) was used to access register-data [14]. The Umeå SIMSAM lab is a data infrastructure where several registers and database sources are included. The register-data covered all people who have lived in Sweden at some point between 1960 to 2017. Intergenerational links were available [14].

The Swedish National Childhood Celiac Disease Register provided data on diagnosed celiac disease before 15 years of age. The diagnose of celiac disease is based on the European Society of Pediatric Gastroenterology, Hepatology and Nutrition (ESPGHAN) guidelines [15]. The register was introduced in 1991, initially covering around 40% of Sweden and became nationwide 1998. Prior to the Umeå SIMSAM lab update to 2.0 the register underwent quality check.

Statistics Sweden provided data from the National Agency for Education Pupil Register including information about school outcomes, and the Longitudinal Integrated Database for Health Insurance and Labor Market studies (LISA database) which provided data about socioeconomic position and demography. Individual data about migration was provided by Statistics Sweden. The National Board of Health and Welfare provided data from the Medical Birth Register which includes information about pregnancy and neonatal health [14]. Only variables motivated by the research questions and ethically approved were provided.

The Umeå SIMSAM Lab infrastructure, including the National Swedish Childhood Celiac Disease Register, has been approved by the Research Ethics Committee of Umeå University (EPN 2018/99–31). The National Swedish Childhood Celiac Disease Register has been ethically approved (dnr 4741–92 and dnr 370–97). Participants in the National Swedish Childhood Celiac Disease Register and their legal guardian have approved participation. All register data is anonymized.

### Study population

**Fig. 1:**
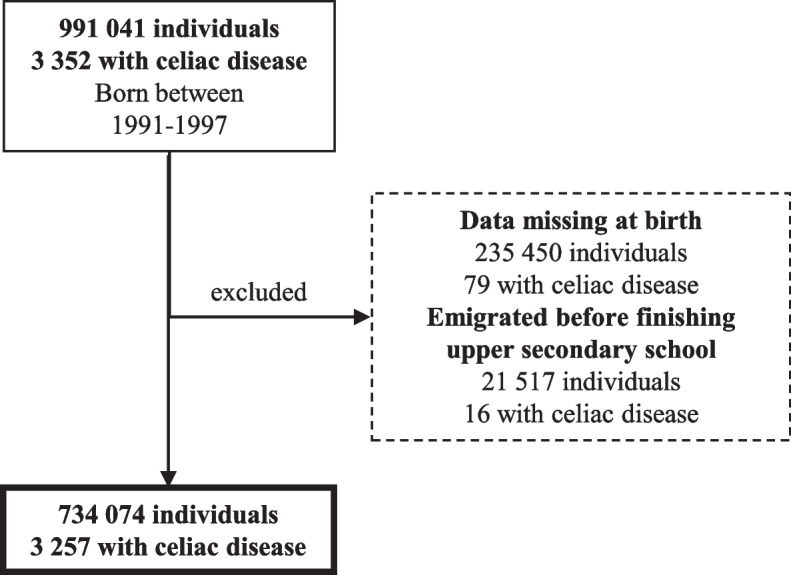
Study population The figure illustrates the initial population and the study population after exclusion

Our study included all children born between 1991–1997 and registered as living in Sweden at some point between their birth and 2017. Everyone that had missing data at birth (for instance all born outside Sweden) or had emigrated from Sweden before finishing upper secondary school were excluded (Fig. 1). Celiac disease diagnosed before 15 years of age, as defined by inclusion in the Swedish National Childhood Celiac Disease Register was considered the main exposure of interest. Before entering the study, we performed a quality control of the Swedish National Childhood Celiac Disease Register which resulted in two celiac disease cases becoming reclassified as non-celiac disease. In total, the study included 734 074 individuals of whom 3 257 had celiac disease.

### School achievements

Outcomes were 1) choice of upper secondary school program, 2) completion of upper secondary school, 3) achievements of basic eligibility for college/university, and 4) final grade in upper secondary school. Outcomes were assessed at the age of 20 years since most Swedish adolescents have finished upper secondary school at that age.

The upper secondary school consist of different educational programs, which were categorized into; 1) university preparing programs, 2) vocational preparing programs, and 3) remaining programs. Remaining programs includes national recruitment programs and private school programs which can be both university- and vocational preparing programs, and introduction programs.

Both completion of upper secondary school and achievement of basic eligibility for college/university were dichotomized (did or did not).

The final grade ranged between 0–20 (lowest 0 and highest 20). In Sweden, the grading system was changed between those born 1994 and 1995, from a four-graded scale to a six-graded scale. Still, the total grading score is interpreted equally.

### Covariates

Based on previous research on factors which have been associated with both celiac disease, health, and school achievements covariates were chosen. We included both individual covariates for the child and family characteristics. Individual characteristics included sex, year of birth, living region at 17 years of age, Apgar score at five minutes, small for gestational age, low birth weight, and age at celiac disease diagnosis. Sex was organized as female and male. Year of birth ranged from 1991–1997 and was categorized into 1991–1994 and 1995–1997, according to changes made in the grading system in Sweden. Apgar score at five minutes is a measure for newborn physical condition five minutes after birth and was categorized according to standard criteria which is low if below seven and normal between seven to ten [16]. Small for gestational age was dichotomized into yes or no pursuant to international standards regarding weight-based growth. Infants are classified as small for gestational age if the birth weight is in the lowest tenth percentile at a certain pregnancy length [17]. Low birth weight was dichotomized into yes or no according to international standards, low birth weight is a weight < 2 500 kg [18]. Age at celiac disease diagnose was categorized into zero to two years of age, three to five years of age, six to twelve years of age, and > 12 years of age. Living region at 17 years of age was categorized according to NUTS one (Nomenclature des Unités Territoriales Statistiques one) which in Sweden is east-, south-, and north part of the country.

Educational level for mother and father were organized into the highest education of the mother or the father. Parental education was categorized into compulsory school ≤ 9 years or upper secondary school, university studies < 3 years, and university studies ≥ 3 years (including Ph.D. studies). Parental income was described as the total yearly income after tax for both parents together. The income was categorized into three categories (low-, average- and high income). Low was everyone in the lowest quartile and high was everyone in the highest quartile. Average was those in-between. The categorizes for parental income were calculated for each year, respectively, thereby taking inflation into account.

### Statistical analyses

Statistical analyses were done using IBM SPSS Statistics for Windows, Version 26.0. Armonk, NY: IBM Corp and were done in the Umeå SIMSAM lab. Descriptive statistics were used to describe the study population. In our analyses, we considered sex, year of birth, living region at 17 years of age, Apgar score at five minutes, small for gestational age, low birth weight, age at diagnose, and parental education and income as potential confounders as previous literature have shown that they might be associated with both celiac disease and educational variables. Associations between celiac disease and these variables were assessed with t-test, analysis of variance (ANOVA) or chi-square test as appropriate. Variables who showed no association with celiac disease, which in our study was Apgar score at five minutes, small for gestational age, and low birth weight, were therefore not included in adjusted analyzed and consequently were sex, birth cohort, living region at 17 years, parental education, and parental income used as covariates in our analyses. Logistic regression was used for dichotomous outcomes, i.e. completion of upper secondary school, and achievements of basic eligibility for college/university. Multiway ANOVA, without interaction terms, was used for our continuous outcome variable upper secondary school final grade, in adjusted analyses. Analyzes were performed stratified on sex.

## Results

### Characteristics of the study population

Almost two thirds of the celiac disease population were females (Table 1). A total of 894 (27%) were diagnosed with celiac disease from zero to two years of age, 313 (10%) at three to five years of age, 1 445 (44%) at six to twelve years of age, and 605 (19%) was diagnosed after twelve years of age. There was no statically significant association between perinatal conditions and celiac disease, except fewer had an Apgar score below seven (Table 1). A total of 51.8% in the celiac disease population lived in the south part of Sweden, which is more than the non-celiac population where it was 43.8% (*p* < 0.001). We found higher parental education and income in the celiac disease population compared to the non-celiac disease population (Table 1).

**Table 1:** Characteristics of the study population

Characteristics	Celiac disease population, *n* (%)	Non-celiac disease population, *n* (%)	*P*-value
**Population**	3 257 (0.4)	730 817 (99.6)	
**Sex**			
Male	1 225 (37.6)	375 618 (51.4)	
Female	2 032 (62.4)	355 199 (48.6)	< 0.001
**Year of birth**			
1991 to 1994	1 997 (61.3)	455 632 (62.3)	
1995 to 1997	1 260 (38.7)	275 185 (37.7)	0.225
**Living region at 17 years**			
East part of Sweden	1 084 (33.3)	270 073 (37.2)	
South part of Sweden	1 683 (51.8)	317 760 (43.8)	
North part of Sweden	485 (14.9)	137 492 (19.0)	< 0.001
**Apgar score 5 min**			
7 till 10	3 215 (98.7)	716 196 (98.0)	
< 7	42 (1.3)	14 621 (2.0)	0.004
**Small for gestational age**			
No	2 141 (89.1)	655 121 (90.0)	
Yes	262 (10.9)	72 689 (10.0)	0.135
**Low birth weight**			
No	2 316 (96.3)	697 133 (95.7)	
Yes	89 (3.7)	31 643 (4.3)	0.123
**Parental education**			
Compulsory school ≤ 9 years or Upper secondary school	1 525 (46.9)	365 658 (50.2)	
University studies < 3 years	670 (20.6)	141 928 (19.5)	
University studies ≥ 3 years (including Ph.D.)	1 054 (32.4)	221 329 (30.4)	0.001
**Parental total income**			
Low	631 (20.3)	172 171 (25.0)	
Average	1 644 (52.7)	343 835 (50.0)	
High	842 (27.0)	171 947 (25.0)	< 0.001

### Upper secondary school program and completion

No statically significant difference was found in choice of upper secondary school program between the celiac disease- and the non-celiac disease population (Table 2). A total of 11.3% in the celiac disease population did not complete upper secondary school compared to 12.5% in the non-celiac disease population (*p* = 0.034). Similarly, less individuals with celiac disease did not achieve basic eligibility for college/university compared to the non-celiac disease population (8.2% versus 9.5%, *p* = 0.024). Celiac disease was neither associated with completion nor achieving basic eligibility for college/university when analyses were adjusted for sex, year of birth, living region at 17 years, parental education, and parental income (Table 3 and 4).

**Table 2:** Upper secondary school programs, completion of upper secondary school, and achievements of basic eligibility for college/university

	**Celiac disease population,***n* (%)	**Non-celiac disease population,n** (%)	*P*-value
**Upper secondary school program**			
University preparing programs	1 325 (45.8)	289 500 (45.3)	
Vocational preparing programs	870 (30.1)	200 455 (31.3)	
National recruitment programs, Introduction programs, and specialized/private school—which could be both university and vocational preparing programs	695 (24.1)	149 506 (23.4)	0.336
**Completion of Upper secondary school**			
No	367 (11.3)	91 354 (12.5)	
Yes	2 890 (88.7)	639 463 (87.5)	0.034
**Achieved basic eligibility for college/ university**			
No	238 (8.2)	60 513 (9.5)	
Yes	2 652 (91.8)	578 950 (90.5)	0.024

**Table 3:** Association with not completed upper secondary school. Table illustrates logistic regression. Outcome is not completed upper secondary school

	**Crude** **(CI 95%)**	**Model one** **(CI 95%)**	**Model two** **(CI 95%)**
**Celiac disease**			
No	Ref	Ref	Ref
Yes	0.89 (0.80–0.99)	1.04 (0.90–1.19)	1.06 (0.96–1.19)
**Sex**			
Female	Ref	Ref	Ref
Male	1.31 (1.29–1.33)	1.34 (1.32–1.36)	1.32 (1.30–1.35)
**Year of birth**			
1991–1994	Ref	Ref	Ref
1995–1997	1.03 (1.01–1.04)	1.06 (1.05–1.08)	1.07 (1.05–1.08)
**Living region at 17 years**			
East part of Sweden	Ref	Ref	Ref
South part of Sweden	0.90 (0.89–0.92)	0.83 (0.81–0.84)	0.83 (0.81–0.84)
North part of Sweden	1.02 (1.00–1.04)	0.94 (0.92–0.96)	0.94 (0.92–0.96)
**Parental education**			
University studies ≥ 3 years (including PhD)	Ref	Ref	Ref
University studies < 3 years	1.29 (1.26–1.32)	1.15 (1.12–1.18)	1.17 (1.14–1.20)
Compulsory school ≤ 9 years or Upper secondary school	2.69 (2.64–2.74)	2.07 (2.02–2.11)	2.07 (2.03–2.12)
**Parental income**			
High	Ref	Ref	Ref
Average	2.01 (2.00–2.05)	1.66 (1.62–1.70)	1.66 (1.62–1.71)
Low	4.15 (4.05–4.25)	3.22 (3.14–3.30)	3.24 (3.16–3.33)
**Apgar score 5 min**			
Apgar 7–10	Ref	Ref	
Apgar < 7	2.36 (2.27–2.45)	1.26 (1.19–1.32)	
**Small for gestaional age**			
No	Ref	Ref	
Yes	1.37 (1.34–1.40)	1.24 (1.21–1.27)	
**Low birth weight**			
No	Ref	Ref	
Yes	1.91 (1.85–1.96)	1.24 (1.19–1.28)

**Table 4:** Association with not achieved basic eligibility for college/university. Table illustrates logistic regression. Outcome is not achieved basic eligibility for college/university

	**Crude (CI 95%)**	**Model one (CI 95%)**	**Model two (CI 95%)**
**Celiac disease**			
No	Ref	Ref	Ref
Yes	0.86 (0.75–0.98)	0.92 (0.78–1.09)	0.94 (0.82–1.08)
**Sex**			
Female	Ref	Ref	Ref
Male	1.24 (1.22–1.26)	1.26 (1.24–1.28)	1.25 (1.23–1.27)
**Living region at 17 years**			
East part of Sweden	Ref	Ref	Ref
South part of Sweden	0.90 (0.88–0.91)	0.85 (0.83–0.87)	0.85 (0.83–0.87)
North part of Sweden	0.90 (0.88–0.93)	0.85 (0.83–0.87)	0.85 (0.83–0.87)
**Parental education**			
University studies ≥ 3 years (including PhD)	Ref	Ref	Ref
University studies < 3 years	1.10 (1.08–1.13)	1.03 (1.00–1.06)	1.03 (1.00–1.06)
Compulsory school ≤ 9 years or Upper secondary school	1.68 (1.65–1.71)	1.40 (1.37–1.43)	1.40 (1.37–1.43)
**Parental income**			
High	Ref	Ref	Ref
Average	1.45 (1.42–1.49)	1.33 (1.30–1.37)	1.33 (1.30–1.37)
Low	2.26 (2.20–2.32)	1.99 (1.94–2.05)	2.00 (1.94–2.05)
**Year of birth**			
1991–1994	Ref	Ref	Ref
1995–1997	0.59 (0.57–0.60)	0.59 (0.58–0.60)	0.59 (0.58–0.60)
**Apgar score 5 min**			
Apgar 7–10	Ref	Ref	
Apgar < 7	1.04 (0.98–1.11)	1.02 (0.96–1.10)	
**Small for gestaional age**			
No	Ref	Ref	
Yes	1.12 (1.09–1.15)	1.11 (1.08–1.14)	
**Low birth weight**			
No	Ref	Ref	
Yes	1.06 (1.02–1.11)	1.00 (0.95–1.04)	

### Final grade from Upper secondary school

**Fig. 2:**
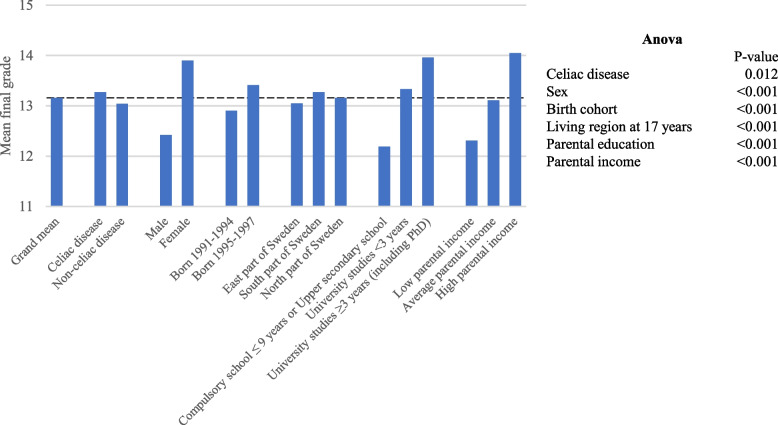
Adjusted mean final grade. The figure present ANOVA main effects. Upper secondary school mean final grade is the outcome. Adjusted mean final grade is illustrated for covariates

**Fig. 3:**
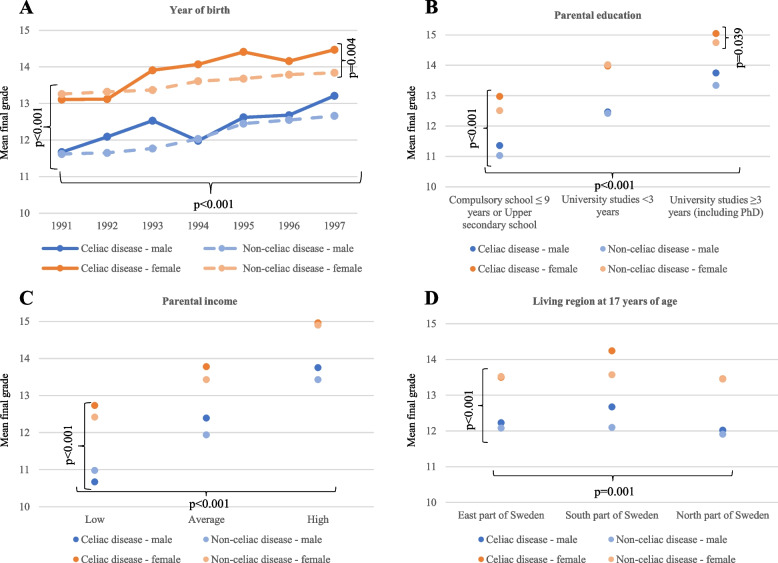
Stratified mean final grade A indicates mean final grade depending on year of birth separated into sex and celiac disease diagnose. B indicates mean final grade depending on parental education separated into sex and celiac disease diagnose. C indicates mean final grade depending on parental income separated into sex and celiac disease diagnose. D indicates mean final grade depending on living region at 17 years of age separated into sex and celiac disease diagnose

The mean final grade in the celiac disease population was 13.34 (standard deviation (SD) 4.85). This was significantly higher than the non-celiac population (12.78 SD 5.01, *p* < 0.001, Supplementary material table A). Mean final grade was not affected by the age at celiac disease diagnosis when stratifying for birth cohorts (born 1991–94 *p* = 0.217 and born 1995–97 *p* = 0.968). Both females and males with celiac disease had a statically significant higher mean final grade than their peers without celiac disease (Supplementary material table A). The effect from celiac disease on mean final grade remained when adjusting for sex, birth cohort, living region at 17 years, and parental education and income (*p* = 0.012) (Fig. 2). When adjusting for year at birth, females with celiac disease had higher mean final grade than females without celiac disease (*p* = 0.004, Fig. 3). The same pattern was seen when parental education (0.039) was considered (Fig. 2). There was no difference in females with or without celiac disease when parental income (*p* = 0.060) and living region at 17 years (*p* = 0.098) were considered separately (Fig. 3). Males with celiac disease and without celiac disease did not differ in mean final grade when year at birth (*p* = 0.059), living region at 17 years (*p* = 0.111), parental education (*p* = 0.086) and parental income (*p* = 0.353) were considered separately (Fig. 3). Apgar score at five minutes, small for gestational age and low birth weight did not contribute to the statistical model for the mean final grade in the celiac disease population (Supplementary material, table A). There were few from the celiac disease population with low Apgar score (Table 1).

## Discussion

In this register-based retrospective cohort study we showed that the adolescents with celiac disease diagnosed during childhood received similar or slightly better mean final grade from upper secondary school compared to the non-celiac disease adolescents born during the same period (1991–1997). The adolescents with celiac disease chose similar upper secondary school programs and completed upper secondary school to the same extent as other adolescents. Achievements in upper secondary school in Sweden affects future possibilities in life for example eligibility and competitiveness when applying to higher education, career prospects or when looking for work.

Previous research has investigated socioeconomic position and celiac disease which have shown inconsistent results. A Swedish study did not find any association with socioeconomic position and childhood onset celiac disease [19]. Another Swedish study showed that low socioeconomic position was associated with celiac disease in boys [20]. However, these studies did not investigate if celiac disease caused socioeconomic consequences. Namatovu et al. investigated childhood onset celiac disease and school achievements in ninth grade in a Swedish population and did not find any association [10] which is in line with our findings. On the other hand, socioeconomic consequences in terms of increased work loss both before and after diagnosis have been shown in adults with celiac disease [21].

It is previously known that health is an important factor for achievements in school. School achievement can be affected through several ways such as impaired cognitive and executive function, concentration problems, stress, and absenteeism [7–9]. Celiac disease increases the risk for development of depression, anxiety, and headache [22], which all have been suggested to negatively affect school achievements [23, 24]. Furthermore, celiac disease has been associated with cognitive impairment [25, 26], which could be related to learning difficulties. While risk for co-morbidity decreases with treatment, patients with celiac disease experience a high treatment burden and impact on quality-of-life, often caused by stigma and difficulties to adhere to a gluten-free diet [27–30]. The adherence rate to the gluten-free diet in Sweden is high (86–98%) but adolescence constitutes a period in life when both involuntary and voluntary dietary lapses are more common [5].

Adolescents with celiac disease daily face difficulties with the social situation when dining out with friends, difficulties with increased risk for mental illness, and difficulties with the transition from adolescents to adulthood [30]. Unexpectedly, we found that adolescents with celiac disease had similar or slightly better grades from upper secondary school compared to other adolescents. Why celiac disease did not affect school achievements in upper secondary school is therefore unclear. Lichtwark et al. showed that cognitive impairment in celiac disease improved on a gluten-free diet [25]. In Sweden follow-up in pediatric patients is every year to every second year at the pediatric department where adherence to a gluten-free diet and co-morbidity are determined [29]. A Swedish study showed that follow-up occurs according to recommendations [31]. All celiac disease cases in this study were diagnosed before 15 years of age, meaning that they had received their diagnosis before entering upper secondary school, had time from their diagnosis and should have received follow-up in the pediatric care for at least three years. Our result could therefore suggest that those with diagnosed celiac disease manage their disease and treatment, at least when supported through regular follow-up. If this is the case the importance of follow-up within health care beyond pediatric care is stressed.

In this study the celiac disease population received comparable final mean grade to the non-celiac disease population even when potential confounders were considered. When performing stratified analysis, only females with celiac disease performed a higher mean final grade. The reason why females with celiac disease perform better than their healthy peers can only be hypothesized. Previous research has associated high degree of personal responsibility, measured as a high internal locus of control, to high level of treatment adherence among children with celiac disease [32]. High internal locus of control has been associated with higher school achievements [33]. It could be that the requirement of strict adherence to a gluten-free diet, and the self-management this entails, reinforces traits which lead to better academic achievement. Of note, however, is that hypervigilance to a gluten-free diet can be concerning and extreme vigilance is associated with lower quality-of-life [34].

Although the Umeå SIMSAM Lab is a unique data infrastructure with data recorded prospectively and independently through administrative authorities’ control, we recognize some limitations. In this study population there were few from the celiac disease population with low Apgar score, even though the study population covered whole Sweden and included 3 257 individuals with celiac disease. Apgar score at five minutes, small for gestational age and low birth weight did not affect the outcomes on school achievement in the celiac disease population (Supplementary material, table A) and were therefore not included in the adjusted analyses. Apgar score and other factors from birth are previously known to be associated with lower school achievements [35]. Due to limited power, we could not investigate this further. An inherent limitation of the register-based studies is lack of data not routinely collected. In our study it would have been interesting to have individual data about adherence to the gluten-free diet and follow-up. However, Swedish studies indicates high adherence in adolescents and follow-up according to recommendations [5, 31]. The National Swedish Childhood Celiac Disease Register became nationwide 1998. Therefore, individuals who were diagnosed between 1991–1998, and not residents of the included areas during those years were not included. We have assessed the impact and found no significant effect. Inflation in school grades found in the study, might have resulted in an overestimation of final grades, thus they should be interpreted with caution. This study is performed in the Swedish school system. Thus, we cannot be certain that our findings can be generalized to other countries since a multitude of factors within both school- and health care systems may affect school achievements. Still, we believe that our findings are applicable to at least well-adherent adolescents who attend a follow-up program.

## Conclusions

In conclusion, we found that celiac disease diagnosed during childhood does not negatively affect school achievements in upper secondary school in Sweden. In this study celiac disease was diagnosed before 15 years of age. Thus, the majority had lived with celiac disease for several years prior to upper secondary school and should have received follow-up medical care at the pediatric department. Our findings therefore suggest that diagnosis, adherence to a gluten-free diet and follow-up programs can minimize potential negative effects of the celiac disease on school achievements at upper secondary school. Future studies are necessary to determine if other school and health care systems have an effect and if socioeconomic consequences appear later in life.

## Supplementary Information

**Additional file 1. **

## Data Availability

The datasets generated and/or analysed during the current study are not publicly available because the Swedish Data Protection Act (1998:204) does not permit sensitive data on humans to be freely shared. The data can be accessed after application and approval from the steering board of the Umeå SIMSAM lab (https://www.umu.se/forskning/infrastruktur/umea-simsam-lab/). After approval, the data will be retrieved and stored in a folder accessible from computers in the Umeå SIMSAM lab. The Umeå SIMSAM lab is located in Umeå, Sweden.

## Abbreviations

Umeå SIMSAM lab: The Swedish Initiative for Research on Microdata in the Social and Medical Sciences 2.0
ESPGHAN: The European Society of Pediatric Gastroenterology, Hepatology and Nutrition
LISA database: The Longitudinal Integrated Database for Health Insurance and Labor Market studies
NUTS one: Nomenclature des Unités Territoriales Statistiques one
ANOVA: Analysis of variance
SD: Standard deviation
