# Supplementary material for: Celiac disease and upper secondary school achievement in Sweden A retrospective cohort study
Source: BMC Pediatr. 2022 Dec 12;22:709. doi: 10.1186/s12887-022-03773-6 (PMC9743674; doi:10.1186/s12887-022-03773-6)
Supplement: Supplementary file 1 — Additional file 1. [file 12887_2022_3773_MOESM1_ESM.docx]

| **Characteristics** | **Mean grade  celiac disease population (SD) n=3357** | **Mean grade  non-celiac disease population (SD) n=730 817** | **P-value** |
| --- | --- | --- | --- |
| **Total population** | 13.34 (4.85) n=2 890 | 12.78 (5.01) n=639 463 | <0.001 |
| **Sex** |  |  |  |
| Male | 12.43 (4.85) n=1 061 | 12.06 (4.96) n=323 338 | 0.015 |
| Female | 13.88 (4.77) n=1 829 | 13.53 (4.95) n=316 125 | 0.003 |
| P-value | <0.001 | <0.001 |  |
| **Living region at 17 years** |  |  |  |
| East part of Sweden | 13.04 (5.05) n=958 | 12.79 (5.16) n=236 764 | 0.141 |
| South part of Sweden | 13.66 (4.63) n=1 505 | 12.83 (4.94) n=282 035 | <0.001 |
| North part of Sweden | 12.92 (5.05) n=427 | 12.67 (4.84) n=120 284 | 0.280 |
| P-value | 0.001 | <0.001 |  |
| **Apgar score 5min** |  |  |  |
| 7 till 10 | 13.34 (4.86) n=2 853 | 12.79 (5.01) n=628 467 | <0.001 |
| <7 | 13.57 (3.50) n=37 | 12.64 (5.04) n=10 996 | 0.259 |
| P-value | 0.773 | 0.002 |  |
| **Small for gestational age** |  |  |  |
| No | 13.51 (4.76) n=1 924 | 12.81 (4.99) n=576 029 | <0.001 |
| Yes | 13.77 (4.59) n=219 | 12.59 (5.13) n=61 174 | <0.001 |
| P-value | 0.444 | <0.001 |  |
| **Low birth weight** |  |  |  |
| No | 13.52 (4.77) n=2 072 | 12.79 (5.01) n=612 846 | <0.001 |
| Yes | 14.07 (3.63) n=73 | 12.61 (5.06) n=25 073 | 0.014 |
| P-valúe | 0.323 | <0.001 |  |
| **Year of birth** |  |  |  |
| 1991 to 1994 | 13.05 (5.18) n=1 767 | 12.56 (5.31) n=399 172 | <0.001 |
| 1995 to 1997 | 13.81 (4.23) n=1 123 | 13.15 (4.44) n=240 291 | <0.001 |
| P-value | <0.001 | <0.001 |  |
| **Parental education** |  |  |  |
| Compulsory school ≤ 9 years or Upper secondary school | 12.41 (4.74) n=1 299 | 11.76 (5.04) n=303 267 | <0.001 |
| University studies <3 years | 13.42 (4.94) n=609 | 13.21 (4.73) n=129 192 | 0.258 |
| University studies ≥3 years (including PhD) | 14.55 (4.65) n=976 | 14.03 (4.80) n=205 625 | <0.001 |
| P-value | <0.001 | <0.001 |  |
| **Parental total income** |  |  |  |
| Low | 11.95 (5.50) n=507 | 11.69 (5.35) n=137 423 | 0.289 |
| Average | 13.28 (4.62) n=1 464 | 12.68 (4.82) n=306 370 | <0.001 |
| High | 14.51 (4.46) n=803 | 14.15 (4.59) n=162 065 | 0.028 |
| P-value | <0.001 | <0.001 |  |

P-value<0.05 indicates a statistical significant difference.
n= total number
SD= Standard deviation

**Supplementary material
Table A.** Mean final grade
Table present mean final grade from upper secondary school for each covariate separated into the celiac disease population and the non-celiac disease population.
